# Systematic Development of an Intervention to Promote Self-Sampling for HIV and Sexually Transmitted Infections for Men Who Have Sex With Men: An Intervention Mapping Approach

**DOI:** 10.3389/frph.2021.634032

**Published:** 2021-05-10

**Authors:** Jeanine Leenen, Christian J. P. A. Hoebe, Arjan E. R. Bos, Petra F. G. Wolffs, Inge H. M. van Loo, John B. F. de Wit, Kai J. Jonas, Nicole H. T. M. Dukers-Muijrers

**Affiliations:** ^1^Department of Sexual Health, Infectious Diseases, and Environmental Health, South Limburg Public Health Service, Gemeentelijke Geneeskundige Dienst (GGD) Zuid Limburg, Heerlen, Netherland; ^2^Department of Medical Microbiology, Care and Public Health Research Institute (CAPHRI), Maastricht University Medical Centre (MUMC+), Maastricht, Netherland; ^3^Department of Social Medicine, Care and Public Health Research Institute (CAPHRI), Maastricht University Medical Centre (MUMC+), Maastricht, Netherland; ^4^Faculty of Psychology, Open University, Heerlen, Netherland; ^5^Department of Interdisciplinary Social Science, Utrecht University, Utrecht, Netherland; ^6^Centre for Social Research in Health, University of New South Wales, Sydney, NSW, Australia; ^7^Department of Work and Social Psychology, Faculty of Psychology and Neuroscience, Maastricht University, Maastricht, Netherland; ^8^Department of Health Promotion, Care and Public Health Research Institute (CAPHRI), Maastricht University Medical Centre (MUMC+), Maastricht, Netherland

**Keywords:** MSM, HIV, sti, self-sampling, intervention mapping

## Abstract

Sexual healthcare aims to reduce HIV and sexually transmitted infections (STIs) by promoting testing and prevention. To better reach men who have sex with men (MSM), additional strategies are needed. Here, we describe development of an intervention, which is part of a broader HIV/STI home-care program, targeted to reach MSM and motivate them to use self-sampling tests. Self-sampling includes blood sampling (finger prick) for HIV, hepatitis B, and syphilis, and a urine sample and oral and anorectal swab samples for chlamydia and gonorrhea. Intervention mapping, a systematic six-step approach, was used to guide the development process: (1) needs assessment including interviews with MSM, (2) create a matrix of change, (3) selection of theory-based methods and practical strategies, (4) intervention development, (5) implementation plan, and (6) evaluation (not included in this paper). Stakeholders were involved to increase program support and feasibility. The needs assessment revealed that testing barriers among MSM related to stigma, time, and privacy concerns. Barriers among healthcare providers related to time, competing priorities, lack of expertise, and guideline restrictions. Included intervention components are designed to overcome these barriers, e.g., engaging role models, with a website with a role model story, and providing tailored information. Methods to reach MSM were a variety of information channels (posters, flyers, and audio-visual displays) and delivery modes, such as advertisements on websites and invitational cards (online and paper) distributed by healthcare providers and MSM themselves (social network testing/peer testing). Our intervention aims to encourage MSM to engage in testing, re-testing, and providing a test to peer MSM. Evidence-based methods to overcome barriers were included to reach and motivate an increased number of MSM. Using intervention mapping stimulated systematic evidence-based decision making and adapting the intervention to the target audience and setting. The next step (step 6) is to implement and evaluate the intervention.

## Introduction

Men who have sex with men (MSM) are at increased risk of acquiring human immunodeficiency virus (HIV) and sexually transmitted infections (STIs) (1). In the Netherlands, the majority of newly diagnosed HIV infections are among MSM (2, 3). Among MSM visiting Dutch STI clinics, 0.3% were diagnosed with HIV and 21.2% were diagnosed with an STI (including HIV) in 2019 (2). HIV/STI testing and early linkage to care are critical for improving long-term individual health outcomes (4, 5). Lack of (timely) HIV/STI testing introduces concerns for individual health, as well as public health concerns, in terms of ongoing transmission and adverse health outcomes. To reduce the number of new HIV and STI infections, timely testing, treatment, and prevention is key. International guidelines for HIV/STI management recommend that all sexually active MSM are tested at least annually for HIV and all other relevant STIs: syphilis, hepatitis B (HBV), and anorectal, genital, and oropharyngeal infections of *Neisseria gonorrhoeae* (NG) and *Chlamydia trachomatis* (CT) (1, 6). In the Netherlands, MSM can get tested at sexual healthcare centers (STI clinics), which deliver comprehensive sexual healthcare including free-of-charge testing for HIV, HBV, syphilis, and (extra) genital bacterial STI, STI treatment, HIV care referral to the hospital HIV treatment center, partner notification, and sexual health counseling. MSM can also get tested at the general practitioner (GP) for HIV and STIs.

However, in spite of numerous public health efforts to promote HIV/STI testing and prevention, many MSM are not reached with high-quality care and remain untested or not regularly tested; this leaves HIV/STI infections untreated (2, 3). In the Netherlands, among MSM visiting STI clinics, only 18.9% test every 6 months for HIV/STI (7). The statistics in our region (South Limburg, the Netherlands) are as follows: 28% of all MSM never tested before, 67% of young (<25 years) MSM never tested before, and only 20% tested regularly at the STI clinic (8). Several barriers to getting tested among MSM have been identified in previous studies, including expected stigma from healthcare providers or laboratory staff who the MSM would need to see, fear of the potential consequences of a positive test result, lack of time to attend care, privacy concerns such as fear of being recognized at the STI clinic by other people, low-risk perception, and lack of motivation to be tested (9–13).

Therefore, additional care strategies to reach MSM with HIV/STI tests need to be explored. Alternatives to face-to-face clinic testing include (1) self-testing, where MSM collect their specimen, perform a test, and interpret the test result in private, or (2) self-sampling, where MSM collect their specimen in private and send their specimens to a laboratory where they are tested, and the laboratory returns the test result for HIV and STIs (14). Self-collected samples may include urine, blood, or saliva, or an oropharyngeal or anorectal swab. Reliable self-tests, showing direct test results, are available for HIV but are lacking for other STIs. Self-sampling (thus with accurate laboratory testing) can be performed at a person's home, which is also referred to as home-sampling. Self-sampling for urogenital and for extragenital NG and CT is comparable with clinician-administered samples (15–17).

Self-sampling for HIV/STI at home, additional to clinic-based testing, is expected to improve test uptake in MSM, has the potential to reach people who would otherwise not get tested, and serves as an entry point into HIV/STI prevention and care (18–21). Self-sampling can potentially overcome barriers posed by in-clinic, face-to-face testing, such as fear of being seen at the clinic, expected stigma or long waiting times (21, 22). Self-sampling in the home setting could work especially well in more rural regions, as people living in these areas may face environmental barriers, such as transportation constraints (23). Home-based HIV/STI testing was found acceptable and convenient by MSM and can make testing easy, and time- and place-independent (24, 25). Self-sampling tests were also found acceptable by healthcare providers, as they acknowledged the speed of home-sampling, the reduced workload of staff, and more privacy and confidentiality toward the patients when using self-sampling (18, 26, 27). Although, the potential benefits of self-sampling have been well-described, home-sampling has not been widely implemented in regular sexual healthcare and is currently not widely used by MSM. A recent study among MSM in Europe showed that knowledge of the existence of self-sampling for HIV is relatively low (25%), and actual use is very low (1%) (28). During the COVID-19 pandemic, with the closing or more limited availability of physical testing locations, the demand for care at home has surfaced even more (29, 30). Regional data from China and Australia indicated that the number of MSM undergoing facility-based HIV testing reduced by more than half during the COVID-19 pandemic (30, 31).

Systematically designing a healthcare program to promote healthy behavior (here: getting tested for HIV/STI) is essential for an effective implementation and wide uptake of the healthcare program. An intervention developed based on theory and evidence, and together with the target group and implementers of the intervention, is likely to be effective and sustainable (32, 33). Intervention mapping (IM) is a health promotion protocol for selecting and applying social and behavioral science theories, to the planning, implementation, and evaluation of health promotion interventions (33). Disease prevention interventions that have used IM have generally reported significant increases in the uptake of disease prevention programs (34). Intervention mapping is a six-step approach that can be used to guide a systematic development of interventions based on theory and evidence. In step 1, a needs assessment is performed. In step 2, performance and change objectives are formulated based on knowledge gained in step 1. In step 3, theory-based intervention methods are selected to create change of the determinants of the behavior. In step 4, program components and specific materials are selected. In step 5, a plan for implementation of the program is made. Step 6, the implementation and evaluation, is beyond the scope of this article and will be described separately, after the intervention has been implemented.

The aim of this study is to describe the systematic development, according to the IM protocol, of an evidence-based intervention to reach MSM, and encourage them to undergo regular testing for HIV/STI with self-collected home-sampling tests. This intervention is part of a broader new home-care program that provides MSM with high-quality home-sampling tests for HIV and STIs and sexual healthcare.

## Materials and Methods

The intervention described in this paper is part of a broader home-care program for MSM. This home-care program includes a kit with sampling materials for (1) blood sampling (used for HIV, syphilis, and hepatitis B testing), (2) urine sampling (used for genital NG and CT testing), and (3) extragenital sampling (swabs used for oropharyngeal and anorectal NG and CT testing). The sampling kits are returned by postal mail for laboratory testing. The home-care program is integrated in existing healthcare networks between different sexual healthcare providers and aims to engage MSM in HIV/STI testing, treatment, and care. This program also addresses sexual health and prevention strategies, such as condom use, PrEP and partner notification, and active follow-up for individuals who test positive for HIV/STI to ensure they are engaged in care. The program is regional-focused and is designed for the province of Limburg, Southwest of the Netherlands. As part of the home-care program, we developed a strategy (the intervention) to promote the use of self-sampling tests. The framework for intervention development will be described in this section, and the application of this framework to design the intervention components is described in the **Results** section.

Before the development of the intervention, a planning group of 12 people was established. A brief stakeholder assessment was done to explore who is potentially affected by the intervention and which stakeholders and experts from different disciplines and sectors should be included in the planning group. The planning group consisted of public health experts from the STI clinic of the public health service South Limburg, who were the initiators of the intervention, behavior change experts, and other stakeholders (decision-makers and potential intervention implementers) in care practice. The stakeholders included STI clinic and HIV treatment center care providers (nurses and physicians), a GP, laboratory staff, and STI clinic policy makers. Stakeholders were involved to increase support, feasibility, and success of the intervention (33, 35). Public health experts were responsible for the intervention's development and will oversee the implementation of the intervention in a later stage. Healthcare providers shared their expertise on the target population, practice, and feasibility of new ideas. Behavior change experts shared their expertise on psychology and behavior change. Together, the planning group set a shared goal of promoting HIV/STI testing among MSM living in the area of study (region of Limburg) in the Netherlands who do not get regularly tested. Regular meetings with group members were held to discuss possible strategies to achieve the project goal and development progression.

### Step 1: Needs Assessment (Logic Model of the Problem)

The first step of IM, a needs assessment, identifies the problem and determines what personal and environmental factors are related to the problem. In this article, we address the personal factors associated with MSM who do not (regularly) test for HIV/STI. Step 1 provides clarity on what should be changed and what the context for the intervention is, such as the population and setting. We conducted a literature search on the setting in which the intervention will be implemented, barriers preventing MSM from getting tested for HIV/STI, and on the methods that facilitate testing (self-sampling). Results from the literature search are embedded in the introduction.

Next, semi-structured, face-to-face interviews lasting 20–50 min were conducted among MSM to identify specific barriers to HIV/STI testing and to determine an effective and feasible method to enhance self-sampling HIV/STI tests. Interviewers were trained and guided prior to and during administering interviews by the same supervisor. Eighteen MSM of 18 years and older (mean age was 32 years) were recruited from a STI clinic (GGD Zuid Limburg) and Dutch lesbian, gay, bisexual, and transgender support organization (COC Maastricht) based on convenience sampling. Participants were asked to provide their ideas and opinions about self-sampling at home, and the logistic procedure of these tests, such as receiving and returning the tests and on stimulating others to use self-sampling tests. Interviews were analyzed using qualitative analysis methods and QualiCoder software (*Under development*—Greater Good, Maastricht, the Netherlands). Statements that were mentioned more than once by participants were identified as a code. The codes were grouped into broader thematic concepts, such as attitudes, perceived barriers, preferences, experiences, personal ideas, outcome expectations, and self-efficacy of participants toward home-sampling test for HIV/STI. Ethical approval for the interviews was obtained from the Ethical Committee Psychology of Maastricht University (ERCPN 04_09_2012_S6). Written consent was obtained from the participants before the start of the interview.

### Step 2: Intervention Outcomes and Objectives (Logic Model of Change)

After the intervention goal was specified, objectives were formulated at the behavioral level to achieve the goal of MSM getting tested. These specific objectives are called performance objectives (PO). Then, for each PO, the behavioral determinants derived from theoretical frameworks were selected based on importance (contribution to the behavior) and changeability, which was assessed by the planning group. A principled system of importance of themes was used by the planning group, guided by the current STI/HIV diagnosis and treatment guidelines for STI/HIV sexual healthcare in our country. Selection of determinants was guided by literature, information gained from interviews (the needs assessment), and expert advice from the planning group. Finally, the PO and their behavioral determinants were combined to create matrices of change objectives (COs), to identify what should be targeted by the intervention. Each CO was formulated to be measurable and action-oriented.

### Step 3: Intervention Design

Using the results of the needs assessment in step 1 and the matrix of change created in step 2, theory-based intervention methods were selected to address the determinants selected in step 2 to promote behavior change.

For each determinant and CO, a theoretical method for influencing changes in the behavioral determinants was selected. Methods were selected based on literature and expert advice of the planning group members, taking into account the target population, feasibility, and changeability of the determinants. Behavior change experts shared their opinions and experiences regarding the methods that could be used to effectively promote behavior change.

Next, every method was converted into a practical application, which is a specific technique for the practical use of theoretical methods in ways that fit with the target group and the context in which the intervention will be conducted. The experts from the planning group converted these methods into practical applications by taking into account the target group, parameters for effectiveness of the selected methods, and the results of the needs assessment performed in step 1 (33). STI clinic and HIV treatment center care providers shared their opinions and experiences regarding the applications that were likely to be feasible and fit the target group.

### Step 4: Intervention Production

The practical applications were combined into intervention components. Again, both implementers (care providers) and representatives of the target group (MSM) of the intervention were involved and consulted in the process. Members of the planning group were asked to share their opinions and experiences during brainstorm meetings to select the best possible intervention design for the target group, to increase the likelihood that the intervention would be used properly and would reach the set goal. This step also involves an assessment of whether the intervention components and materials will be feasible in terms of time and budget constraints and if the target group will be reached.

### Step 5: Implementation Plan

In the Netherlands, MSM can be tested for HIV/STI at STI clinics, GP offices, and HIV treatment centers. Therefore, the intervention was intended to be implemented by healthcare providers at these locations. Public health experts, who are responsible for the development of the intervention, also guided and oversaw the implementation of the intervention. To maximize implementation, key stakeholders were engaged in the development and implementation process.

In order to prepare for implementation of the intervention, information regarding the implementers' needs and barriers to the delivery and promotion of HIV/STI testing among MSM was first gathered through an exploratory literature search, expert opinion, and semi-structured face-to-face interviews with 19 healthcare providers from the STI clinic of the public health service South Limburg and HIV treatment center Maastricht. Participants of the interview were potential implementers of the intervention and were included based on convenience sampling. With the information obtained from this needs assessment, a plan was made for adoption, implementation, and management of the intervention in real-life contexts.

## Results

### Step 1: Needs Assessment

The needs assessment was carried out in two steps: a literature search and additional qualitative research. The results from the literature search are embedded in the introduction. Results showed that not all MSM are regularly tested for HIV/STI. Barriers for testing were mainly related to expected stigma, time, and privacy concerns. Self-sampling tests have been proven feasible and effective for increasing HIV/STI testing.

The qualitative research consisted of interviews with members of the target group. The interviews with MSM (*n* = 18) showed that self-collected home-sampling tests are considered to be highly acceptable. Home-sampling was judged as highly promising by MSM to overcome main barriers to getting tested. Interviewed MSM stated that these tests were perceived as time-efficient, reliable, convenient, easy, modern, and innovative. MSM mentioned that it was important that the sampling could be conducted in the comfort of their own homes. Consequently, they appreciated not having to make the effort to travel to the health clinic and not having to wait for appointments. Participants appreciated that the self-sampling tests would lead to savings in money for gas and parking. Furthermore, some participants valued that they would not have to take time off from work and could use the sampling kit whenever it suited them best. Participants explained that these positive aspects increased the likelihood of them making the effort to get a HIV/STI test and therefore expected that they and possibly other MSM would use self-sampling if available. Several participants stated that they did not like sampling blood sample, which would be a possible barrier to use self-sampling. Furthermore, some participants were afraid that the sampling could be wrongly executed and thereby influencing the reliability of the tests. Also, concerns toward the lack of face-to-face counseling were raised.

**Figure 1:**
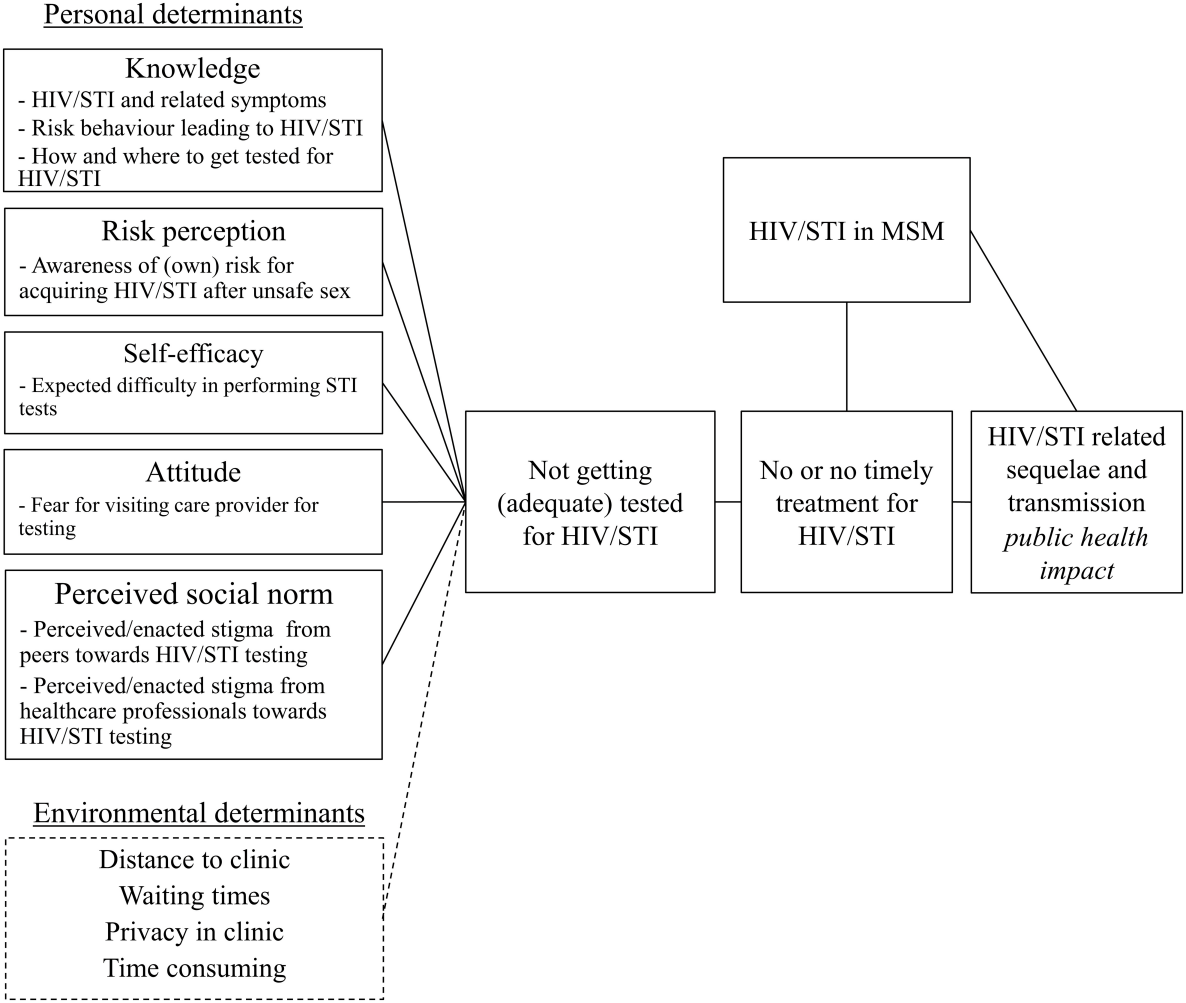
Logic model of the problem regarding lack of HIV/STI testing among men who have sex with men.

Based on the findings from step 1, the needs assessment, a logic model of the problem was created (Figure 1) and the following program goal was formulated: MSM are tested using self-sampling for all relevant HIV/STI at all three relevant anatomic sites at least twice a year.

### Step 2: Program Outcomes and Objectives

In order to reach the program goal that MSM get themselves tested for all relevant HIV/STI at all three relevant anatomic sites using self-sampling tests, six POs were formulated (Table 1). First, MSM decides to get tested (PO1). Second, MSM requests a sampling kit (PO2). Third, MSM performs sampling according to the instructions (PO3). Fourth, MSM should return the sampling kit (*via* postal mail) (PO4). If test results are positive, MSM should make an appointment for treatment (PO5), and complies with treatment (PO6). For each PO, determinants were selected. Determinants included knowledge, risk perception, self-efficacy/skills, attitude (cognitive/affective), and perceived social norms. For example, the most important and changeable determinants for PO1 were knowledge, risk perception, and perceived social norms (Table 1).

**Table 1:** Performance and change objectives to promote HIV/STI testing with self-sampling tests among men who have sex with men (MSM).

**Performance objectives**	**Determinants**				
	**Knowledge**	**Risk perception**	**Self-efficacy/skills**	**Attitude (cognitive/affective)**	**Perceived social norm**
PO1. MSM decides to get tested.	K1. Describe when it is necessary to go for HIV/STI testing.	R1. Appraise personal risks of HIV/STI after unprotected sex.			SN1. Acknowledge responsibility for own and partners' health.
PO2. MSM requests a sampling kit.	K2. State how and where you can order a sampling kit.	R2. Appraise effects of HIV/STI and personal risk.	SE1. Express confidence in ability to order a sampling kit.	• A1. Acknowledge the advantages of using the sampling kit. • A2. Acknowledge the importance of getting tested.	
PO3. MSM uses sampling kit correctly according to the instructions.	K3. State how to perform blood and urine sampling and how to perform extragenital swab sampling.		• SE2. Express confidence in ability to perform the test. • SK1. Able to demonstrate how to perform the test. • SK2. Able to formulate ways of dealing with negative emotions involved with testing.	• A3. Acknowledge emotions involved with testing (i.e., fear) • A4. Acknowledge the pros and cons of testing.	
PO4. MSM returns sampling kit *via* mail.	K4. List where and how you can return a sampling kit.			A5. Acknowledge the emotions involved in the outcome (i.e., fear of a positive test).	
PO5. MSM makes an appointment for treatment.	K5. Describe how to get treatment.			A6. Acknowledge the importance of getting treatment.	SN2. Acknowledge responsibility to get treated for HIV/STI.
PO6. MSM complies with treatment.	K6. Describe the treatment procedure.		SE4. Express confidence in complying with treatment.		SN3. Acknowledge responsibility to comply with the treatment protocol (i.e., no unsafe sexual contact during treatment).

Next, for every PO and their determinants, a CO was formulated. For example, for PO3, “MSM uses sampling kit correctly according to the instructions.” CO SE2, “Express confidence in ability to perform the test,” was formulated to address the determinant self-efficacy. A matrix of change was made (Table 1).

### Step 3: Program Design

For each determinant selected in step 2, change objectives were formulated. For example, for the determinant knowledge, change objective K1 (“Describe when it is necessary to go for HIV/STI testing”), the method “tailoring” was chosen. When using tailoring, the intervention (components) will be adjusted to previously measured characteristics of participants of the target group. The method “tailoring,” chosen to change the determinant knowledge about HIV/STI and HIV/STI testing, was transformed into a practical application of providing tailored information to fit the MSM target group by presenting healthcare information only relevant for MSM. Other examples of selected methods and applications are displayed in Table 2.

**Table 2:** Examples of selected theoretical methods and applications used to get men who have sex with men (MSM) tested for HIV/STIs.

**Determinant**	**Change objective**	**Parameters for use**	**Method**	**Application**
Knowledge	K1. Describe when it is necessary to go for HIV/STI testing.	Tailoring variables to relevance.	Tailoring	Information tailored to target group provided on printed flyers/posters.
	K3. State how to perform blood and urine sampling and how to perform extragenital swab sampling.	Schematic guides of what is to be learned.	Advance organizers	Schematic test instruction with images on how to perform sample collection.
Risk perception	R1. Appraise personal risks of HIV/STI after unprotected sex.	Cognitive and affective appraisal of self-image.	Self-reevaluation	High-risk behavior for acquiring a HIV/STI is described on flyer and poster.
	R2. Appraise effects of HIV/STI and personal risk.	Present messages as individual and undeniable.	Personalize risk	Website contains a short questionnaire on risky sexual behavior, generating personal feedback on HIV/STI risk.
Self-efficacy/skills	SK1. Demonstrate how to perform the test.	Credible source Attention and identification with the model	Verbal persuasion Role models	Video instructions performed by role model on how to perform self-collected test.
Attitude (cognitive/affective)	A1. Acknowledge the advantages of using the sampling kit.	Attention and identification with the model.	Role models	Role model story on website about self-sampling experience.

For each determinant and change objective, a theoretical method was selected. For example, self-efficacy is constructed from Social Cognitive Theory (36–38). Perceived self-efficacy refers to beliefs in one's capabilities to organize and execute the courses of action required to achieve the desired change. Unless people believe they are able to achieve desired changes by their actions, they have little incentive to act or to persevere in the face of difficulties and setbacks. A way of creating and strengthening self-beliefs of efficacy is through experiences provided by social models (39).

Risk perception (perceived susceptibility), for example, comes from the Health Belief Model (HBM) (40, 41) and refers to the belief about the chances of being exposed to a certain health outcome, for example, being infected with HIV/STI (40). The HBM theory suggests that people's beliefs about health problems, perceived benefits and barriers of action, and self-efficacy explain (lack of) health-promoting behavior. Interventions based on the HBM may aim to increase risk perception and perceived seriousness of a health condition by providing education about prevalence and incidence of disease, individualized estimates of risk, and information about the medical, financial, and social consequences of disease (40). The HBM contains several concepts that predict why people will take action to prevent, to screen for, or to control illness conditions; these include susceptibility, seriousness, benefits, and barriers to a behavior, cues to action, and self-efficacy (40). A construct of the HBM is a stimulus, or cue to action, which must be present in order to trigger the health-promoting behavior. Therefore, in our intervention, we incorporated multiple stimuli in different stages in our intervention to promote MSM to use home-sampling to test for HIV/STI.

### Step 4: Intervention Production

The main intervention components developed by the planning group were a website, cards with an invitation to be tested, and different information channels [posters, flyers, and narrowcasts (audio-visual displays)].

To increase knowledge and awareness, different information channels for MSM were developed [posters, flyers, and narrowcast (audio-visual display)]. Development was accomplished together with representatives of the target group and healthcare providers, in order for the intervention to fit the target group. MSM stated in the interviews that information on self-sampling tests should be displayed not only at specific venues for MSM (such as gay sauna's) but also in public places. Therefore, posters, flyers, and narrowcast will be displayed in relevant and healthcare departments (e.g., waiting areas at GPs' offices, schools, HIV treatment centers, and STI clinics) and contain short information on the importance of HIV/STI testing and a link to a website where sampling kits can be ordered. The posters, flyers, and narrowcast will serve as a stimulus to HIV/STI testing and to visit the website. Promotional materials will contain information on risk factors for acquiring HIV/STI, addressing the determinant “knowledge,” in order to accomplish K1 (“Describe when it is necessary to go for HIV/STI testing”), which is a change objective from PO1 (“Decides to get tested”).

Previous studies have consistently shown that a person's self-efficacy and intention to test are key determinants of actual testing (42). A person with low testing intention is unlikely to return the tests when test packages are just given to him or her. Therefore, in our intervention, MSM will receive cards with an invitation to order a self-sampling kit online. Subsequently, MSM have to perform the action of going to a specifically designed website to order a sampling kit. On the website, there will be eligibility questions for MSM who want to order a test (for example, MSM can only request a test when residing in the region served by the implementing healthcare providers). MSM are also asked to fill in questions for care and evaluation purposes. Interviewed MSM stated that it would acceptable to go through these steps, as the free-of-charge home-sampling tests they subsequently receive will make this worth the effort. This website contains several elements that will increase MSM's self-efficacy and testing intention. The website will contain information about HIV/STI and how MSM can order a sampling kit. This website will also address testing barriers and will contain several methods to promote behavior change. First, information on the website about risk factors, HIV/STI, and testing presented on this website will be tailored to the needs of MSM (method: tailoring). Second, there will be a short story of a male role model and his personal positive experience with home-sampling tests (method: role models). MSM stated in the interviews that a role model expressing a positive experience with home-sampling would encourage them to use a home-sampling test. Engaging MSM themselves as role models in HIV prevention strategies and peer-led intervention has a greater improvement in knowledge of HIV and improving behavioral outcomes (43, 44).

In cases where MSM have ordered and received their sampling kit, but did not return it within 2 weeks, up to three text message reminders will be sent. The STI clinic will send tailored SMS reminders. Reminders (text messages) will be used to promote testing behavior, as reminders have proven to increase participation (45, 46). The text messages will also give MSM the opportunity to contact the STI clinic if they want more information or have questions (for example, about HIV/STI, self-sampling tests, drug use during sex: chemex or PrEP). Interviewed MSM were concerned about the lack of face-to-face counseling; therefore, these text messages will give MSM the opportunity to contact healthcare providers in an accessible way.

To reach MSM who are not reached by healthcare providers or by the information channels used in the intervention (online advertisement and narrowcasts or as posters and flyers), an additional method (i.e., including the social network) was chosen to further facilitate HIV/STI testing by mobilizing social support. All MSM who use a self-sampling kit will be invited (*via* website, healthcare provider, etc.,) to also offer a testing opportunity to their MSM peers. Sexual network characteristics are related to interconnectedness and concurrency of sex partners, facilitating HIV/STI spread. Members of the social and sexual networks surrounding those with (previous) HIV/STI infections are also at high risk of acquiring HIV/STI infections (47). Furthermore, peers are more likely to influence behaviors of other MSM in their social network than professionals and thereby may be better able to connect to members of their social networks who are not linked to care. Peers and social network testing have therefore the potential to increase HIV/STI detection and testing behavior in networks of young people and MSM (42, 48–51). Offering a HIV/STI self-sampling test has the potential to be awkward, and people who test for HIV/STI may fear being stigmatized by others if they were to disclose sensitive information or to distribute a test. However, interviewed MSM stated that they would be willing to give a test (invitation) to their peers. Also, previous studies have shown that people effectively avoid being stigmatized by disclosing information (52) or distributing tests (42) only to “trusted peers.” The friend is more likely to get tested, because he or she received the test from a role model with a positive experience to testing. Moreover, peers may also consciously or unconsciously select the friends who are more at risk, which would also potentially increase the effectiveness of this method (53). The role model on the website will also address peer-testing and will give some tips to open a conversation with peers about HIV/STI testing and offering the self-sampling test.

As people with a previous STI are more likely to become infected again (7–20%) (54–56), healthcare providers (GPs, providers at HIV treatment centers, and STI clinic nurses and physicians) can actively offer a testing opportunity to MSM patients who previously had a STI. The patients will receive a text message with a link to the website and a unique code to order a home-sampling test.

### Step 5: Implementation Plan

Results of the interviews with implementers of the intervention (healthcare providers from HIV treatment centers and GPs) revealed that barriers in offering and addressing STI tests to MSM were mainly lack of time, competing medical priorities, lack of testing and treatment knowledge (expertise), discomfort with sexual history and genital examinations, patient reluctance, role reluctance (responsibility), and financial or guidelines restrictions (13, 57, 58).

Next, a collaborative infrastructure was established between regional healthcare providers from HIV treatment centers, GPs and STI clinics, implementers, and planning group members that enables and facilitates information. This will support implementation and increase the likelihood of the intervention continuing. Because of its primary public health goal, and its responsibility in delivering high-quality HIV/STI care, the STI clinic is best suited to facilitate, manage, and organize the changes in process of care required from GPs, hospital care providers, and STI nurses and physicians.

The last step of the implementation plan will be to inform key care providers (e.g., heads of departments) about the program in a face-to-face meeting. They will play an active role in implementing the program in their department and in motivating their colleagues to use the program. These key persons serve as the contact person for their colleagues and for the public health experts (creators of the program). When implemented, regular evaluation and feedback meetings will be arranged between the public health experts and key persons in order to keep healthcare providers motivated and involved.

## Discussion

We used IM to develop an intervention to promote HIV/STI self-sampling in MSM. The intervention is systematically based on theory and evidence and designed for practical application by healthcare providers in STI clinics, HIV treatment centers, and GPs' offices, to reach MSM and promote uptake of HIV/STI testing. The intervention consists of cards with an invitation for testing, and different offline and online information channels [posters, flyers, and narrowcasts (audio-visual displays)], a website and text message reminders to promote the use of HIV/STI self-sampling tests. This intervention will be part of a home-care program that combines home-based testing with complete care, offering counseling, treatment, retesting opportunities, and partner notification combined with eHealth in order to improve HIV/STI control in MSM.

The intervention consists of distributing invitations to test for HIV/STI *via* self-sampling kits. Offering a self-sampling testing opportunity yields higher testing rates compared with offering a testing opportunity at a clinic (21, 59). As the invitation for testing will be distributed by various online and offline ways (*via* healthcare providers, social network, and *via* posters flyers and website advertisements), we try to maximize our reach with this intervention. In our intervention, we will employ the social network of an MSM who is currently in care or recently tested. Using the social network gives an opportunity to reach MSM who are not reached with current healthcare and has the opportunity to increase social support and reduce perceived stigma around STI/HIV testing. In a study among black MSM, receiving social support from other black MSM friends was associated with lower risk of delayed HIV testing (51). Social network testing/peer HIV testing outside the healthcare setting is a possible way of increasing uptake of testing in high-risk groups (60).

In our intervention, we use mobile text messages to (1) send reminders for MSM who have received home-sampling tests, but did not complete the test and (2) send an invitation for retesting 6 months after testing positive for HIV/STI. Using mobile text messages is widely recognized as an effective communication method between healthcare professionals and their clients and is increasingly accepted in healthcare setting. Studies with text message reminders have increased HIV/STI re-testing among MSM and other populations (61–63). Mobile text message reminders are a cheap and efficient addition to increase participation and a low-threshold method for MSM to contact healthcare providers if needed.

In the development process, using IM stimulated the identification of specific determinants that influence behavior change. When using a structured protocol, it ensures that all relevant determinants are addressed. Combining expert opinions and theories made it clear which elements and program components would be most effective and should be included in an intervention. We experienced that using the IM protocol as described by the developers is potentially a lengthy process, requiring time (possibly more than what is available in the day-to-day practice) of sexual healthcare providers. This issue with using IM thoroughly and according to textbook instructions is also described by other authors (64, 65). However, IM can also be applied in a condensed version, also serving as a highly valuable guide for development and may in some practical settings be a more feasible option. In our study, we did a condensed version of the needs assessment, interviewed participants were selected based on convenience sampling, and expert advice was gathered in an informal setting. Although, these things could have been performed in a more elaborate structured manner, we adapted this process so it fitted the needs and resources available in our practice-based development of this intervention. The use of both extended and condensed versions of the IM protocol has shown to be useful and effective for development of interventions among several public health domains, such as in promoting sexual health programs, cancer screening, and physical activity (66–69).

In our planning group, we had the advantage of having behavior change and IM experts in our planning group, as sufficient theoretical knowledge and experience with technical IM aspects have to be available. Yet, the IM protocol is written in clear steps; therefore, the protocol can also be used for developers without previous behavioral change theory expertise. The protocol enhances a better understanding of the complexity of a behavior by breaking down behavior in understandable terms of performance objectives and underlying change objectives. Therefore, the use of IM when designing an intervention is feasible in a variety of settings. Using IM ensures that the intervention is adapted to the regional setting in which the intervention will be implemented, but is also useful for adapting an existing program from one population to another.

In our study, using IM facilitated the collaboration between intervention developers, implementers, and stakeholders and between different healthcare providers. This collaboration is a solid base for implementing the intervention and enhances patient sexual healthcare between different disciplines (STI clinic, HIV treatment center, GP, and laboratory staff). Overall, we feel that using IM has benefited our development process by serving as a guide for development, ensuring there was a clear understanding of the problem and its determinants and ensuring there is a solid base for implementation of the intervention. Therefore, we would recommend other developers to use IM or another structured protocol for developing interventions.

## Limitations

In our needs assessment, we interviewed MSM based on convenience sampling due to time and resource restrictions. These MSM where all familiar with sexual healthcare. Although, these interviews give an insight into acceptance and preferences of using self-sampling among MSM, it would also be good to have this information available for MSM who are not familiar with regular sexual healthcare. Therefore, more research is needed on how to reach MSM who are currently not reached with care.

Expert advice was collected in a semi-structured way (i.e., mainly by brainstorm sessions, guided by structured list of themes that were deemed important a priori or were raised *ad-hoc*). Collection of advice was done during several stages of the development process. As the information collection process was mainly informal, it is possible that an over-representation of a subset of perspectives and under-representation of others have occurred. However, as experts were from different disciplines and different backgrounds, different perspectives were taken into account.

This article focused on the personal behavior aspects of testing behavior. Although, environmental conditions can influence health problems directly and indirectly through their influence on behavior, we did not describe determinants at the environmental level. However, with the developed program, several environment-related testing barriers (e.g., distance to the testing clinic and privacy issues in the building) may be mitigated by self-sampling at home (22, 23).

In this article, steps 1–5 of the intervention mapping protocol (development of the intervention) have been described. Step 6, evaluation of the intervention, will be conducted in the next phase. Implementation of the broader home-care program was pilot tested in a hospital setting, which allowed for a process evaluation to optimize the program and its implementation. This pilot focused on sustainable implementation, yielding valuable new scientific insights and practical information (46). The full implementation of the home-care program, including this intervention, is currently ongoing.

## Conclusions

This intervention that promotes HIV/STI self-sampling testing among MSM was systematically developed for effective behavioral change. IM is a useful guide to develop interventions in practice for health promotion. In the program, evidence-based methods to overcome barriers are included to reach an increased number of MSM and motivate healthcare providers. The next step (step 6 of the IM approach) is to evaluate the adopted and implemented program. The clear documentation of the development process of an intervention could be very useful to other public health professionals who are developing healthcare programs.

## Data Availability Statement

The raw data supporting the conclusions of this article will be made available by the authors, without undue reservation.

## Ethics Statement

The studies involving human participants were reviewed and approved by Ethical Committee Psychology of Maastricht University. The patients/participants provided their written informed consent to participate in this study.

## Author Contributions

JL drafted the manuscript and designed the intervention. ND-M supervised the study, designed the intervention, and contributed in the drafting of the manuscript. CH, AB, PW, IL, JW, and KJ participated in designing the intervention and contributed in the drafting of the manuscript. All authors read and approved the final manuscript.

## Conflict of Interest

The authors declare that the research was conducted in the absence of any commercial or financial relationships that could be construed as a potential conflict of interest.

## References

[1] Workowski KA. Centers for disease control and prevention sexually transmitted diseases treatment guidelines. Clin Infect Dis. (2015) 61(suppl. 8):S759–62. 10.1093/cid/civ771 26602614

[2] Staritsky L Van Aar F Visser M Heijne J Götz H Nielen M. Sexually Transmitted Infections in the Netherlands in 2019 Bilthoven: RIVM (2020).

[3] van Sighem A Wit F Boyd A Smit S Matser A Reis P. HIV Monitoring Report: Human Immunodeficiency Virus (HIV) Infection in the Netherlands. Stichting HIV monitoring (SHM) (2020). Available online at: https://www.hiv-monitoring.nl/application/files/7716/0571/6500/Netherlands_HIV_Monitoring_Report_2020.pdf (accessed February 9, 2021).

[4] Rutstein SE Ananworanich J Fidler S Johnson C Sanders EJ Sued O. Clinical and public health implications of acute and early HIV detection and treatment: a scoping review. J Int AIDS Soc. (2017) 20:21579. 10.7448/IAS.20.1.21579 28691435 PMC5515019

[5] Gray RT Prestage GP Down I Ghaus MH Hoare A Bradley J. Increased HIV testing will modestly reduce HIV incidence among gay men in NSW and would be acceptable if HIV testing becomes convenient. PLoS ONE. (2013) 8:e55449. 10.1371/journal.pone.0055449 23457470 PMC3574096

[6] Templeton DJ Read P Varma R Bourne C. Australian sexually transmissible infection and HIV testing guidelines for asymptomatic men who have sex with men 2014: a review of the evidence. Sex Health. (2014) 11:217–29. 10.1071/SH14003 24690473

[7] Visser M Heijne JC Hogewoning AA van Aar F. Frequency and determinants of consistent STI/HIV testing among men who have sex with men testing at STI outpatient clinics in the Netherlands: a longitudinal study. Sex Transm Infect. (2017) 93:396–403. 10.1136/sextrans-2016-052918 28159917 PMC5574382

[8] Slurink I van Aar F Op de Coul E Heijne J van Wees D Hoenderboom B. Sexually Transmitted Infections in the Netherlands in 2018 Bilthoven: RIVM (2019).

[9] Wayal S Llewellyn C Smith H Fisher M. Home sampling kits for sexually transmitted infections: preferences and concerns of men who have sex with men. Cult Health Sex. (2011) 13:343–53. 10.1080/13691058.2010.535018 21154069

[10] Deblonde J De Koker P Hamers FF Fontaine J Luchters S Temmerman M. Barriers to HIV testing in Europe: a systematic review. Eur J Public Health. (2010) 20:422–32. 10.1093/eurpub/ckp231 20123683

[11] Mikolajczak J Hospers HJ Kok G. Reasons for not taking an HIV-test among untested men who have sex with men: an internet study. AIDS Behav. (2006) 10:431–5. 10.1007/s10461-006-9068-8 16501868

[12] Kampman CJ Heijne JC Kistemaker-Koedijk PH Koedijk FD Visser M Hautvast JL. Determinants of frequent and infrequent STI testing and STI diagnosis related to test frequency among men who have sex with men in the eastern part of the Netherlands: a 6-year retrospective study. BMJ Open. (2018) 8:e020495. 10.1136/bmjopen-2017-020495 29858416 PMC5988078

[13] Barbee LA Dhanireddy S Tat SA Marrazzo JM. Barriers to bacterial STI testing of HIV-infected men who have sex with men engaged in HIV primary care. Sex Transm Dis. (2015) 42:590. 10.1097/OLQ.0000000000000320 26372931 PMC4576720

[14] Harding-Esch EM Hollis E Mohammed H Saunders JM. Self-sampling and self-testing for STIs and HIV: the case for consistent nomenclature. Sex Transm Infect. (2017) 93:445–8. 10.1136/sextrans-2016-052841 27811311

[15] Wilson J Wallace H Loftus-Keeling M Ward H Hulme C Wilcox M. 4 Clinician-Taken Extra-Genital Samples for Gonorrhoea and Chlamydia in Women and MSM Compared with Self-Taken Samples Analysed Separately and Self-Taken Pooled Samples. London: BMJ Publishing Group Ltd. (2017). 10.1136/sextrans-2017-053264.64

[16] Wilson J Wallace H Loftus-Keeling M Ward H Hulme C Wilcox M. Self-Taken Extra-Genital Samples Compared with Clinician-Taken Extra-Genital Samples for the Diagnosis of Gonorrhoea and Chlamydia in Women and MSM. London: BMJ Publishing Group Ltd. (2016). 10.1136/sextrans-2016-052718.5

[17] Lunny C Taylor D Hoang L Wong T Gilbert M Lester R. Self-collected versus clinician-collected sampling for Chlamydia and Gonorrhea screening: a systemic review and meta-analysis. PLoS ONE. (2015) 10:e0132776. 10.1371/journal.pone.0132776 26168051 PMC4500554

[18] Flowers P Riddell J Park C Ahmed B Young I Frankis J. Preparedness for use of the rapid result HIV self-test by gay men and other men who have sex with men (MSM): a mixed methods exploratory study among MSM and those involved in HIV prevention and care. HIV Med. (2017) 18:245–55. 10.1111/hiv.12420 27492141 PMC5347967

[19] Patel AV Abrams SM Gaydos CA Jett-Goheen M Latkin CA Rothman RE. Increasing HIV testing engagement through provision of home HIV self-testing kits for patients who decline testing in the emergency department: a pilot randomisation study. Sex Transm Infect. (2019) 95:358–60. 10.1136/sextrans-2018-053592 29903889 PMC6294698

[20] Ogale Y Yeh PT Kennedy CE Toskin I Narasimhan M. Self-collection of samples as an additional approach to deliver testing services for sexually transmitted infections: a systematic review and meta-analysis. BMJ Glob Health. (2019) 4:e001349. 10.1136/bmjgh-2018-001349 31139454 PMC6509609

[21] Shih SL Graseck AS Secura GM Peipert JF. Screening for STIs at Home or in the Clinic? Curr Opin Infect Dis. (2011) 24:78. 10.1097/QCO.0b013e32834204a8 21124216 PMC3125396

[22] Gilbert M Thomson K Salway T Haag D Grennan T Fairley CK. Differences in experiences of barriers to STI testing between clients of the internet-based diagnostic testing service GetCheckedOnline.com and an STI clinic in Vancouver, Canada. Sex Transm Infect. (2018) 95:151–6. 10.1136/sextrans-2017-053325 29437984 PMC6580770

[23] Ohl ME Perencevich E. Frequency of human immunodeficiency virus (HIV) testing in urban vs. rural areas of the United States: results from a nationally-representative sample. BMC Public Health. (2011) 11:681. 10.1186/1471-2458-11-681 21884599 PMC3223880

[24] van Loo IH Dukers-Muijrers NH Heuts R van der Sande MA Hoebe CJ. Screening for HIV, hepatitis B and syphilis on dried blood spots: a promising method to better reach hidden high-risk populations with self-collected sampling. PLoS ONE. (2017) 12:e0186722. 10.1371/journal.pone.0186722 29053737 PMC5650165

[25] Reagan MM Xu H Shih SL Secura GM Peipert JF. A randomized trial of home versus clinic-based STD screening among men. Sex Transm Dis. (2012) 39:842. 10.1097/OLQ.0b013e3182649165 23064532 PMC3476063

[26] Pearson WS Kreisel K Peterman TA Zlotorzynska M Dittus PJ Habel MA. Improving STD service delivery: would American patients and providers use self-tests for gonorrhea and chlamydia? Prevent Med. (2018) 115:26–30. 10.1016/j.ypmed.2018.08.007 30096329

[27] Napierala Mavedzenge S Baggaley R Corbett EL. A review of self-testing for HIV: research and policy priorities in a new era of HIV prevention. Clin Infect Dis. (2013) 57:126–38. 10.1093/cid/cit156 23487385 PMC3669524

[28] Katz DA Golden MR Hughes JP Farquhar C Stekler JD. HIV self-testing increases HIV testing frequency in high-risk men who have sex with men: a randomized controlled trial. J Acquir Immune Defic Syndr. (2018) 78:505–12. 10.1097/QAI.0000000000001709 29697595 PMC6037557

[29] Sanchez TH Zlotorzynska M Rai M Baral SD. Characterizing the impact of COVID-19 on men who have sex with men across the United States in April, (2020). AIDS Behav. (2020) 24:2024–32. 10.1007/s10461-020-02894-2 32350773 PMC7189633

[30] Chow EPF Hocking JS Ong JJ Phillips TR Fairey CK. Sexually transmitted infection diagnoses and access to a sexual health service before and after the national lockdown for COVID-19 in Melbourne, Australia. Open Forum Infect Dis. (2020) 8:ofaa536. 10.1093/ofid/ofaa536 33506064 PMC7665697

[31] Booton RD Fu G MacGregor L Li J Ong JJ Tucker JD. The impact of disruptions due to COVID-19 on HIV transmission and control among men who have sex with men in China. J. Int AIDS Soc. (2021) 24:e25697. 10.1002/jia2.25697 33821553 PMC8022092

[32] Franche R-L Baril R Shaw W Nicholas M Loisel P. Workplace-based return-to-work interventions: optimizing the role of stakeholders in implementation and research. J Occup Rehabil. (2005) 15:525–42. 10.1007/s10926-005-8032-1 16254753

[33] Eldredge LKB Markham CM Ruiter RA Kok G Fernandez ME Parcel GS. Planning Health Promotion Programs: An Intervention Mapping Approach. Hoboken, NJ: John Wiley & Sons (2016).

[34] Garba RM Gadanya MA. The role of intervention mapping in designing disease prevention interventions: a systematic review of the literature. PLoS ONE. (2017) 12:e0174438. 10.1371/journal.pone.0174438 28358821 PMC5373531

[35] Colquhoun HL Squires JE Kolehmainen N Fraser C Grimshaw JM. Methods for designing interventions to change healthcare professionals' behaviour: a systematic review. Implement Sci. (2017) 12:30. 10.1186/s13012-017-0560-5 28259168 PMC5336662

[36] Schunk DH. Social cognitive theory. In: Harris KR Graham S Urdan T McCormick CB Sinatra GM Sweller J, eitors. APA Handbooks in Psychology. APA Educational Psychology Handbook, Vol. 1. Theories, Constructs, and Critical Issues American Psychological Association (2012) p. 101–23.

[37] Bandura A. The evolution of social cognitive theory. In: Smith KG Hitt MA, editors. Great Minds in Management. Oxford: Oxford University Press (2005). p. 9–35.

[38] Bandura A. Social Foundations of Thought and Action. Englewood Cliffs, NJ: Prentice-Hall (1986). p. 23–8.

[39] Bandura A. Health promotion from the perspective of social cognitive theory. Psychol Health. (1998) 13:623–49. 10.1080/0887044980840742231295924

[40] Glanz K Rimer BK Viswanath K. Health Behavior and Health Education: Theory, Research, and Practice. Hoboken, NJ: John Wiley & Sons (2008).

[41] Rosenstock IM Strecher VJ Becker MH. Social learning theory and the health belief model. Health Educ Q. (1988) 15:175–83. 10.1177/109019818801500203 3378902

[42] Theunissen K Hoebe C Kok G Crutzen R Kara-Zaïtri C De Vries N. A web—based respondent driven sampling pilot targeting young people at risk for chlamydia trachomatis in social and sexual networks with testing: a use evaluation. Int J Environ Res Public Health. (2015) 12:9889–906. 10.3390/ijerph120809889 26308015 PMC4555318

[43] Terui S Huang J Goldsmith JV Blackard D Yang Y Miller C. Promoting transformative community change for equitable health: peer education and intervention for pre-exposure HIV prophylaxis. J Health Commun. (2020) 25:191–203. 10.1080/10810730.2020.1730526 32116152

[44] Medley A Kennedy C O'Reilly K Sweat M. Effectiveness of peer education interventions for HIV prevention in developing countries: a systematic review and meta-analysis. AIDS Educ Prevent. (2009) 21:181–206. 10.1521/aeap.2009.21.3.181 19519235 PMC3927325

[45] Desai M Woodhall SC Nardone A Burns F Mercey D Gilson R. Active recall to increase HIV and STI testing: a systematic review. Sex Transm Infect. (2015) 91:314–23. 10.1136/sextrans-2014-051930 25759476

[46] Leenen J Hoebe CJPA Ackens RP Posthouwer D van Loo IHM Wolffs PFG. Pilot implementation of a home-care programme with chlamydia, gonorrhoea, hepatitis B, and syphilis self-sampling in HIV-positive men who have sex with men. BMC Infect Dis. (2020) 20:925. 10.1186/s12879-020-05658-4 33276727 PMC7716461

[47] Drumright LN Frost SD. Rapid social network assessment for predicting HIV and STI risk among men attending bars and clubs in San Diego, California. Sex Transm Infect. (2010) 86(Suppl. 3):iii17–23. 10.1136/sti.2010.045914 20966457

[48] Simoni JM Nelson KM Franks JC Yard SS Lehavot K. Are peer interventions for HIV efficacious? A systematic review. AIDS Behav. (2011) 15:1589–95. 10.1007/s10461-011-9963-5 21598034 PMC3607378

[49] Shangani S Escudero D Kirwa K Harrison A Marshall B Operario D. Effectiveness of peer-led interventions to increase HIV testing among men who have sex with men: a systematic review and meta-analysis. AIDS Care. (2017) 29:1003–13. 10.1080/09540121.2017.1282105 28150501 PMC5570465

[50] Ross MW Larsson M Jacobson J Nyoni J Agardh A. Social networks of men who have sex with men and their implications for HIV/STI interventions: results from a cross-sectional study using respondent-driven sampling in a large and a small city in Tanzania. BMJ Open. (2016) 6:e012072. 10.1136/bmjopen-2016-012072 27864245 PMC5129084

[51] Scott HM Pollack L Rebchook GM Huebner DM Peterson J Kegeles SM. Peer social support is associated with recent HIV testing among young black men who have sex with men. AIDS Behav. (2014) 18:913–20. 10.1007/s10461-013-0608-8 24065436 PMC3965658

[52] Bos AE Kanner D Muris P Janssen B Mayer B. Mental illness stigma and disclosure: consequences of coming out of the closet. Issues Ment Health Nurs. (2009) 30:509–13. 10.1080/01612840802601382 19591025

[53] den Daas C Geerken M Bal M de Wit J Spijker R Op de Coul E. Reducing health disparities: key factors for successful implementation of social network testing with HIV self-tests among men who have sex with men with a non-western migration background in the Netherlands. AIDS Care. (2019) 32:50–6. 10.1080/09540121.2019.1653440 31416354

[54] Hosenfeld CB Workowski KA Berman S Zaidi A Dyson J Mosure D. Repeat infection with Chlamydia and gonorrhea among females: a systematic review of the literature. Sex Transm Dis. (2009) 36:478–89. 10.1097/OLQ.0b013e3181a2a933 19617871

[55] Dunne EF Chapin JB Rietmeijer CA Kent CK Ellen JM Gaydos CA. Rate and predictors of repeat Chlamydia trachomatis infection among men. Sex Transm Dis. (2008) 35:S40–4. 10.1097/OLQ.0b013e31817247b2 18520978

[56] Fung M Scott KC Kent CK Klausner JD. Chlamydial and gonococcal reinfection among men: a systematic review of data to evaluate the need for retesting. Sex Transm Infect. (2007) 83:304–9. 10.1136/sti.2006.024059 17166889 PMC2598678

[57] Carter JW Jr Hart-Cooper GD Butler MO Workowski KA Hoover KW. Provider barriers prevent recommended sexually transmitted disease screening of HIV-infected men who have sex with men. Sex Transm Dis. (2014) 41:137–42. 10.1097/OLQ.0000000000000067 24413496

[58] Dukers-Muijrers NH Somers C Hoebe CJ Lowe SH Niekamp A-ME Lashof AO. Improving sexual health for HIV patients by providing a combination of integrated public health and hospital care services; a one-group pre-and post test intervention comparison. BMC Public Health. (2012) 12:1118. 10.1186/1471-2458-12-1118 23270463 PMC3537529

[59] Wilson E Free C Morris TP Syred J Ahamed I Menon-Johansson AS. Internet-accessed sexually transmitted infection (e-STI) testing and results service: a randomised, single-blind, controlled trial. PLoS Med. (2017) 14:e1002479. 10.1371/journal.pmed.1002479 29281628 PMC5744909

[60] Strömdahl S Hoijer J Eriksen J. Uptake of peer-led venue-based HIV testing sites in Sweden aimed at men who have sex with men (MSM) and trans persons: a cross-sectional survey. Sex Transm Infect. (2019) 95:575–9. 10.1136/sextrans-2019-054007 31113905 PMC6902060

[61] Bourne C Knight V Guy R Wand H Lu H McNulty A. Short message service reminder intervention doubles sexually transmitted infection/HIV re-testing rates among men who have sex with men. Sex Transm Infect. (2011) 87:229–31. 10.1136/sti.2010.048397 21296796

[62] Downing SG Cashman C McNamee H Penney D Russell DB Hellard ME. Increasing chlamydia test of re-infection rates using SMS reminders and incentives. Sex Transm Infect. (2013) 89:16–9. 10.1136/sextrans-2011-050454 22728911

[63] Dukers-Muijrers NH Theunissen KA Wolffs PT Kok G Hoebe CJ. Acceptance of home-based chlamydia genital and anorectal testing using Short Message Service (SMS) in previously tested young people and their social and sexual networks. PLoS ONE. (2015) 10:e0133575. 10.1371/journal.pone.0133575 26230085 PMC4539363

[64] Wolfers ME van den Hoek C Brug J de Zwart O. Using intervention mapping to develop a programme to prevent sexually transmittable infections, including HIV, among heterosexual migrant men. BMC Public Health. (2007) 7:141. 10.1186/1471-2458-7-141 17615052 PMC1947965

[65] Kok G Harterink P Vriens P De Zwart O Hospers HJ. The gay cruise: developing a theory-and evidence-based internet HIV-prevention intervention. Sex Res Soc Policy. (2006) 3:52. 10.1525/srsp.2006.3.2.52

[66] Lamort-Bouché M Sarnin P Kok G Rouat S Péron J Letrilliart L. Interventions developed with the intervention mapping protocol in the field of cancer: a systematic review. Psychooncology. (2018) 27:1138–49. 10.1002/pon.4611 29247578

[67] Mevissen FE van Empelen P Watzeels A van Duin G Meijer S van Lieshout S. Development of Long Live Love+, a school-based online sexual health programme for young adults. An intervention mapping approach. Sex Educ. (2018) 18:47–73. 10.1080/14681811.2017.1389717

[68] Tortolero SR Markham CM Parcel GS Peters RJ Jr Escobar-Chaves SL Basen-Engquist K. Using intervention mapping to adapt an effective HIV, sexually transmitted disease, and pregnancy prevention program for high-risk minority youth. Health Promot Pract. (2005) 6:286–98. 10.1177/1524839904266472 16020623

[69] McEachan RR Lawton RJ Jackson C Conner M Lunt J. Evidence, theory, and context: using intervention mapping to develop a worksite physical activity intervention. BMC Public Health. (2008) 8:326. 10.1186/1471-2458-8-326 18808709 PMC2567979

